# DNase I improves corneal epithelial and nerve regeneration in diabetic mice

**DOI:** 10.1111/jcmm.15112

**Published:** 2020-03-13

**Authors:** Jing Zhang, Yunhai Dai, Chao Wei, Xiaowen Zhao, Qingjun Zhou, Lixin Xie

**Affiliations:** ^1^ Shandong Provincial Key Laboratory of Ophthalmology Shandong Eye Institute Shandong First Medical University & Shandong Academy of Medical Sciences Qingdao China

**Keywords:** corneal wound healing, diabetes mellitus, DNase I, inflammation, oxidative stress

## Abstract

DNase I has been reported to improve diabetic wound healing through the clearance of neutrophils extracellular traps (NETs) caused by neutrophil aggregation. However, the function of DNase I on diabetic corneal wound healing remains unclear. Here, we investigated the effect and mechanism of topical DNase I application on diabetic mouse corneal epithelial and nerve regeneration. Corneal epithelial defects, inflammatory response, regeneration‐related signalling pathways, oxidative stress, corneal innervation and sensation were examined and compared between the diabetic and normal mice. The results confirmed firstly the increased NETs production during the delayed corneal epithelial wound healing of diabetic mice, which was significantly improved through either DNase I or Cl‐amidine administration. Mechanistically, DNase I improved inflammation resolution, reactivated epithelial regeneration‐related signalling pathways and attenuated the accumulation of reactive oxygen species (ROS). Moreover, DNase I application also promoted corneal nerve regeneration and restored the impaired corneal sensitivity in diabetic mice. Therefore, these results indicate that topical DNase I application promotes corneal epithelial wound healing and mechanical sensation restoration in diabetic mice, representing the potential therapeutic approach for diabetic keratopathy.

## 1 INTRODUCTION

The incidence of diabetes mellitus is getting higher year by year in the world, with nearly half a billion people living with different types of diabetes.^1^ As a systemic metabolic disease, serious complications could be developed in various organs or tissues of diabetic patients. In the eye, diabetic complications in ocular surface serve as one of the leading causes of blindness in diabetic patients.^2^ The most predictable pathological changes in diabetic ocular surface include impaired corneal sensation, decreased sub‐basal nerve fibres, delayed epithelial wound healing and superficial punctate keratitis.^3^, ^4^, ^5^ Then, more severe symptoms, such as corneal ulcer, microbial keratitis and even perforation, could be induced on account of the healing dysfunction of corneal wound in diabetic patients.

Previous studies have reported a variety of influence factors that contribute to the impaired regeneration of ocular surface in diabetic patients, which include, but not limited to, increased reactive oxygen species accumulation, reduced corneal nerve innervation and expression disorders of regeneration‐related signallings, such as phosphorylated epidermal growth factor receptor (EGFR), insulin‐like growth factor 1 receptor (IGF‐1R), silent information regulator 1 (Sirt1) and Akt.^6^, ^7^, ^8^, ^9^ Besides, increased inflammatory response is another considerable factor during the regeneration of corneal epithelium and nerve. Neutrophils are the first inflammatory cells migrating to the focal zone during inflammation or tissue damage.^10^, ^11^ One of the defence functions of neutrophils is the formation of neutrophils extracellular traps (NETs), a fibrous network consisting of nuclear and granular components.^12^ The process of NETs formation and NETs release activation is defined as NETosis,^13^, ^14^ and previous studies have revealed that NETosis delays wound healing both in diabetic mice and humans.^15^

It has been generally recognized that DNase families play vital functions in DNA degradation by targeting and shearing DNA sequences. There are two major DNase families, DNase I and DNase II, and multiple enzymes in each family play diverse roles in the development of various diseases.^16^ Among these nucleases, DNase I has the widest description in DNase I family,^16^ and the activity of DNase I has been reported to relate to the occurrence of systemic diseases (like systemic lupus erythematosus),^17^, ^18^, ^19^, ^20^ different organ cancers,^21^, ^22^, ^23^ and inflammatory disease.^24^, ^25^ Previous study have confirmed that DNase I‐dependent NETs degradation was an important treatment for diabetic skin wound.^15^ However, the NETs formation and the effects of DNase I in diabetic corneal epithelial and nerve regeneration remain unclear.

Commonly, the removal of extracellular DNA (eDNA) by DNase I administration and the application of NETs formation‐related PAD4 (a calcium‐dependent enzyme that is key in mediating NETosis) inhibitors (Cl‐amidine) are two major methods for the intervention of NETs formation.^15^, ^26^ However, it still remains unclear about the function and mechanism of DNase I in diabetic corneal wound healing. Thereby, in this present study, we investigated the effects of DNase I and PAD4 inhibitors on corneal epithelial regeneration in diabetic mice and explored the functional mechanism of DNase I in diabetic corneal wound healing.

## 2 MATERIAL AND METHOD

### 2.1 Animals

Male C57BL/6 mice (6‐8 weeks old) were purchased from the Institute of Laboratory Animal Sciences, Chinese Academy of Medical Sciences. All animal experiments were conducted with the approval of the Ethics Committee of Shandong Eye Institute according to the Association for Research in Vision and Ophthalmology Statement for the Use of Animals in Ophthalmic and Vision Research. As described in our previous studies, type 1 diabetes was induced in the mice with intraperitoneal injections of streptozocin (STZ; 50 mg/kg; Sigma‐Aldrich).^27^, ^28^, ^29^ Diabetic mice with a blood glucose level above 16.7 mmol/L at 16 weeks after the final STZ injection were used in this study. For each mouse, only one eye was suffered from the operation of epithelial removal.

### 2.2 Corneal epithelial wound healing

Mice were anaesthetized by an intraperitoneal injection of 0.5 mL 0.9% sodium chloride solution containing 40 μL ketamine and 10 μL chlorpromazine hydrochloride, followed by topical application of proparacaine hydrochloride eye drops (Alcon) in the eye; then, an Algerbrush II remover (Alger Co) was used to scrape the central corneal epithelium (2.75 mm diameter) in mice. Fluorescein staining method was used to examine the residual epithelial defects, and the percentage of the original defect area was calculated with ImageJ software (NIH) as described in our previous studies.^29^, ^30^, ^31^ For DNase I treatment, diabetic mice were topically applied with 1 mg/mL DNase I (5 μL/eye; Roche Diagnostics GmbH) six times a day after the removal of corneal epithelium, whereas age‐matched control mice were topically applied with equal phosphate buffer saline (PBS) as the vehicle control. For PAD4 inhibitor treatment, diabetic mice were intraperitoneal injected Cl‐amidine (10 mg/kg; Absin Bioscience Inc) once a day from 5 days before corneal epithelial injury to 2 days after injury.

### 2.3 Immunofluorescence staining

For immunofluorescence staining, mouse eyeballs were snap frozen with Tissue‐Tek optimum cutting temperature compound (Sakura Finetechnical). Before staining, frozen sections (7 μm) were performed with pre‐treatments including fixation (4% paraformaldehyde), permeabilization (0.1% Triton X‐100) and blocking (10% bovine serum albumin), at room temperature. Then, processed samples were incubated with primary antibodies (showed in Table 1) overnight at 4°C and subsequently incubated with corresponding secondary antibodies for 1 hour at room temperature. Information of primary and secondary antibodies could be addressed to Table 1. Besides, Hoechst 33342 (Solarbio) was used to stain DNA and Eclipse TE2000‐U microscope was used for image capturing.

**Table 1:** Antibodies for immunofluorescence staining and Western blot

Primary antibody	Dilution concentration	Supplier	Code
IGF‐1R	WB (1/1000) IF (1/200)	Abcam	ab182408
Akt	WB (1/1000)	Cell Signaling	4691
*p*Akt	WB (1/1000) IF (1/200)	Abcam	ab66138
Sirt1	WB (1/1000) IF (1/200)	Abcam	ab12193
Ly6G	WB (1/200) IF (1/500)	BD	551459
Anti‐Histone (citrulline R2 R8 R17)	WB (1/1000) IF (1/800)	Abcam	ab5103
Anti‐Histone	WB (1/3000)	Abcam	ab1791
Anti‐PAD4	WB (1/1000)	Abcam	Ab214810
F4/80‐FITC	IF (1/200)	Biolegend	123107
CD206‐PE	IF (1/200)	Biolegend	141705
Anti‐NADPH oxidase 2	WB (1/1000) IF (1/200)	Abcam	ab129068
Anti‐NADPH oxidase 4	WB (1/1000) IF (1/200)	Abcam	ab133303
III β‐tubulin	IF (1/200)	Biolegend	657403
Alexa Fluor 488 goat anti‐rabbit IgG	IF (1/200)	Beyotime	A0423
IF 555 goat anti‐rat IgG	IF (1/200)	Sungene	GR201G‐37C

Abbreviations: IF, immunofluorescence; WB, Western blotting.

### 2.4 Western blot

Total corneal protein was used for the detection of Western blot, which was extracted by lysis in radio immunoprecipitation assay (RIPA) buffer containing a proteinase inhibitor cocktail. Then, the extracted protein samples (10‐20 µg total proteins) were run on 10% or 12% SDS‐PAGE gels and transferred to polyvinylidene fluoride (PVDF) membranes (Millipore). The protein‐loaded membranes were incubated with primary antibodies (showed in Table 1) overnight at 4°C and then followed by the incubation of horseradish peroxidase (HRP)‐conjugated secondary antibody (Amersham Biosciences) for 1 hour at room temperature. The target proteins were developed by the application of enzyme‐linked chemiluminescence (ECL) kit (Pierce), and ImageJ software was used for the quantification of immunoreactive bands.

### 2.5 Enzyme‐linked immunosorbent assay

Mouse corneas were harvested at 48 hours after epithelial removal, and total corneal proteins were extracted with PBS. After centrifugation, sample supernatants were prepared for quantitative sandwich immunoassay detection with enzyme‐linked immunosorbent assay (ELISA) kits, including myeloperoxidase (MPO; JEB) and neutrophil elastase (NE; JEB) according to the manufacturers’ procedures. Absorbance was read with a microplate reader (Molecular Devices) at 450 nm with a reference wavelength of 570 nm.

### 2.6 Isolation and quantification of eDNA

Mouse corneas were harvested at 48 hours after epithelial removal, and eDNA was extracted with phenol (Sigma‐Aldrich).^15^ Then, the NanoDrop One spectrophotometer (Thermo Fisher Scientific) was used for measuring eDNA.

### 2.7 Reverse transcription quantitative polymerase chain reaction

For reverse transcription (RT) quantitative polymerase chain reaction (qPCR), total RNA kits (TaKaRa) were used to extract the total RNA from the whole cornea according to the instructions supplied by manufacturer. Complementary DNAs were synthesized using the PrimeScript First Strand cDNA Synthesis Kit (TaKaRa). SYBR® Green PCR reagents and the Applied Biosystems 7500 Real‐Time PCR System were used for the implementation of real‐time PCR. Primer sequences for RT‐qPCR were showed in Table 2.

**Table 2:** Primer sequences for RT‐qPCR

	Accession number	Forward primer	Reverse primer
iNOS	NM_010927 . 3	TGTCTGCAGCACTTGGATCAG	AAACTTCGGAAGGGAGCAATG
Arginase‐1	NM_007482 . 3	TGGGTGACTCCCTGCATATCT	TTCCATCACCTTGCCAATCC
IL‐12	NM_008351 . 2	GGGACCAAACCAGCACATTG	TACCAAGGCACAGGGTCATCA
IL‐10	NM_010548 . 2	TGCTAACCGACTCCTTAATGCA	TTCTCACCCAGGGAATTCAAA
CD86	NM_019388 . 3	CAGCACGGACTTGAACAACC	CTCCACGAAACAGCATCTGA
CD206	NM_008625 . 2	TTCAGCTATTGGACGCGAGG	GAATCTGACACCAGCGGAA
TNF‐α	NM_013693 . 3	AGCCGATGGGTTGTACCTTG	ATAGCAAATCGGCTGACGGT
MCP‐1	NM_011333 . 3	CAGCAAGATGATCCCAATGAGTAG	TTTTTAATGTATGTCTGGACCCATTC
β‐Actin	NM_007393	ACTGCCGCATCCTCTTCCT	TCAACGTCACACTTCATGATGGA

### 2.8 Corneal reactive oxygen species

For the observation of intracellular ROS generation, fresh corneal cryostat sections were incubated with 10μM fluorescence probe 2,7‐dichlorodihydrofluorescein diacetate and acetyl ester (DCHF‐DA; Molecular Probes) for 30 minutes at room temperature and visualized under an Eclipse TE2000‐U microscope (Nikon).

### 2.9 Measurement of corneal mechanical sensitivity

Corneal mechanical sensitivity was measured by using a Cochet‐Bonnet esthesiometer (Luneau Ophtalmologie) without anaesthesia according to our previous studies.^28^, ^31^ The test began with the maximal length (6 cm) of nylon filament in the esthesiometer and shortened by 0.5 cm each time until we found the corneal touch threshold (which triggered the eye blink response). The longest filament length with a positive blink response was identified as the value of corneal sensitivity.

### 2.10 Corneal whole‐mount staining

Whole‐mount corneas were fixed with Zamboni stationary liquid (Solarbio) for 1 hour; then, the cornea was dissected along the scleral‐limbal region and blocked by PBS with 0.1% Triton X‐100, 2% goat serum and 2% bovine serum albumin for 2 hour. Subsequently, Alexa Fluor 488‐conjugated neuronal class III β‐tubulin mouse monoclonal antibody was used for the incubation of whole‐mount corneas overnight at 4°C. Eclipse TE2000‐U microscope was used for the examination of nerve morphology and image capturing, and ImageJ software was used to calculate the density of the corneal sub‐basal nerve fibres.

### 2.11 Data analysis

All data in this study were obtained from at least three independent experiments, and results are presented as the means ± standard deviation (SD). Comparisons between two groups were performed using the Student t test, and a *P* value of less than .05 was used to indicate the statistical significance.

## 3 RESULTS

**Figure 1:**
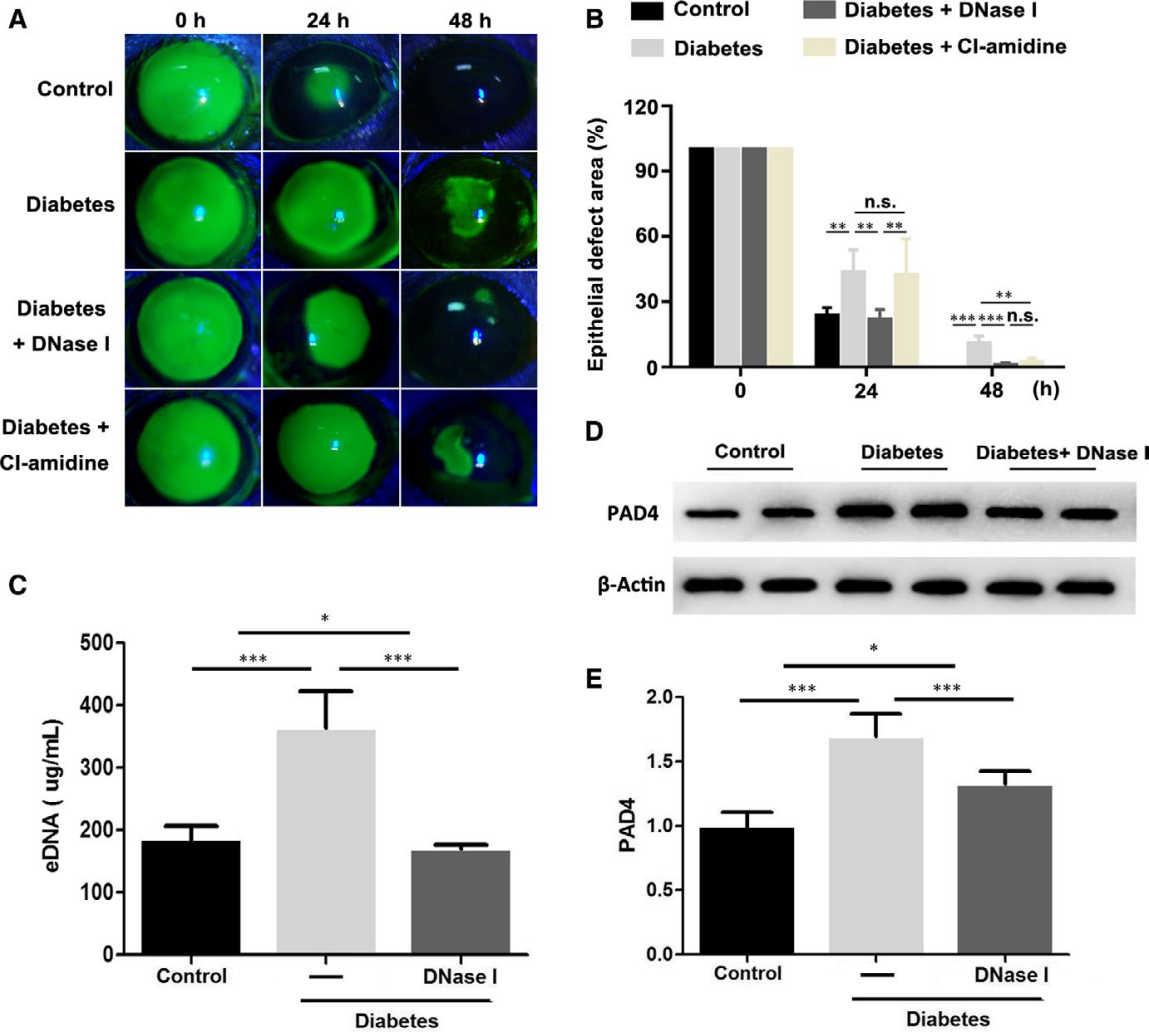
Anti‐NETs treatment promoted the regeneration of corneal epithelium in diabetic mice. A, Diabetic mice were topically treated with 1 mg/mL DNase I (5 μL/eye, six times per day) after the removal of the corneal epithelium. Meanwhile, healthy and diabetic control mice were topically treated with PBS. The residual epithelial defect was examined at 0, 24 and 48 h after the removal of the corneal epithelium with fluorescein staining. B, The histogram of the residual epithelial defect was presented as the percentage of the original wound area (n = 5). C, Corneas harvested 48 h after injury were homogenized and examined for levels of eDNA with spectrophotometer (n = 4). D‐E, Corneas harvested 48 h after injury were evaluated with Western blot to examine the protein contents of PAD4 (n = 6). Data were given as the mean ± SD; **P < .01, ***P < .001, n.s, not significant

To assess the effects of DNase I on the regeneration of corneal epithelium, 1 mg/mL DNase I eye drops was administered to diabetic mice after the removal of corneal epithelium. Similar to previous studies, the regeneration rate of corneal epithelium delayed in diabetic mice, whereas DNase I application efficiently rescued the regeneration rate of corneal epithelium in diabetic mice (Figure 1A). Analysis results of residual epithelial defects showed a remarkable promotion of corneal epithelial regeneration by topical application of DNase I in diabetic mice at 24 and 48 hours after epithelial removal (24 hours: 23.6% ± 3.7% in healthy mice, 43.4% ± 10.5% in diabetic mice, 21.7% ± 4.7% in DNase I‐treated diabetic mice; 48 hours: 0% in healthy mice, 10.9% ± 3.3% in diabetic mice, 0.9% ± 1.0% in DNase I‐treated diabetic mice, Figure 1B; n = 5). Besides, even there is no significant effect of Cl‐amidine application on the healing rate of diabetic corneal epithelium at 24 hours after injury (42.2% ± 16.7%), significant acceleration of corneal epithelial healing rate occurred at 48 hours in diabetic mice (2.1% ± 2.0%). Our results also showed that DNase I not only reduced eDNA content in the cornea of diabetic mice (Figure 1C; n = 4), but also inhibited PAD4 expression (Figure 1D,E; n = 6).

**Figure 2:**
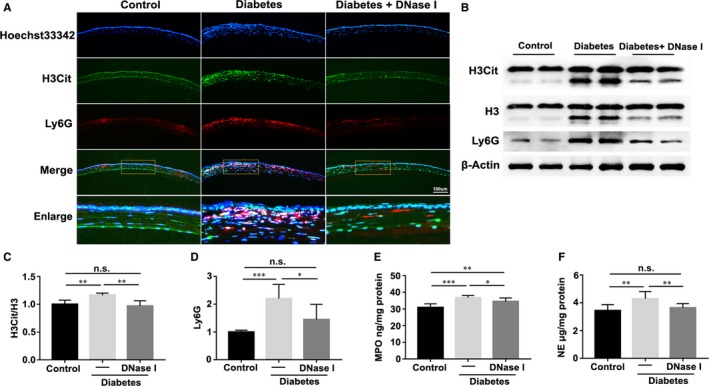
DNase I restored the resolution of corneal inflammation. A, Expression of H3Cit and Ly6G was examined with immunofluorescence staining 48 h after corneal epithelial removal in the control, diabetic and DNase I‐treated diabetic mice. B‐D, Corneas harvested 48 h after injury were evaluated with Western blot to examine the protein contents of H3Cit, H3 and Ly6G (B), accompanied by the quantified results of Western blot experiments (C‐D; n = 6). E‐F, Corneas harvested 48 h after injury were homogenized and examined for levels of myeloperoxidase (MPO) activity (E) and neutrophil elastase (NE) expression (F) with enzyme‐linked immunosorbent assay (ELISA; n = 5). Data were given as the mean ± SD; *P < .05, **P < .01, ***P < .001, n.s, not significant

The infiltration levels of pro‐inflammatory cells were examined after the removal of corneal epithelium, in order to assess the function of DNase I on inflammation resolution in corneal epithelial regeneration. Next, we tested the NET biomarkers, H3Cit, eDNA, MPO and NE. Immunofluorescence staining results revealed the enhanced staining of H3Cit and Ly6G (a neutrophil marker) in corneal stroma in diabetic mice compared with age‐matched healthy mice, whereas topical application of DNase I alleviated the infiltration of neutrophils in diabetic corneal stroma 48 hours after the injury (Figure 2A). Likewise, Western blot results also showed the sufficient function of DNase I in suppressing the overexpressions of H3Cit/H3 and Ly6G in diabetic corneas 48 hours after injury (Figure 2B, C; n = 6). Besides, the ELISA results revealed that the overexpressions of MPO and NE in diabetic corneas were inhibited by DNase I application at 48 hours after injury (Figure 2D; n = 5).

**Figure 3:**
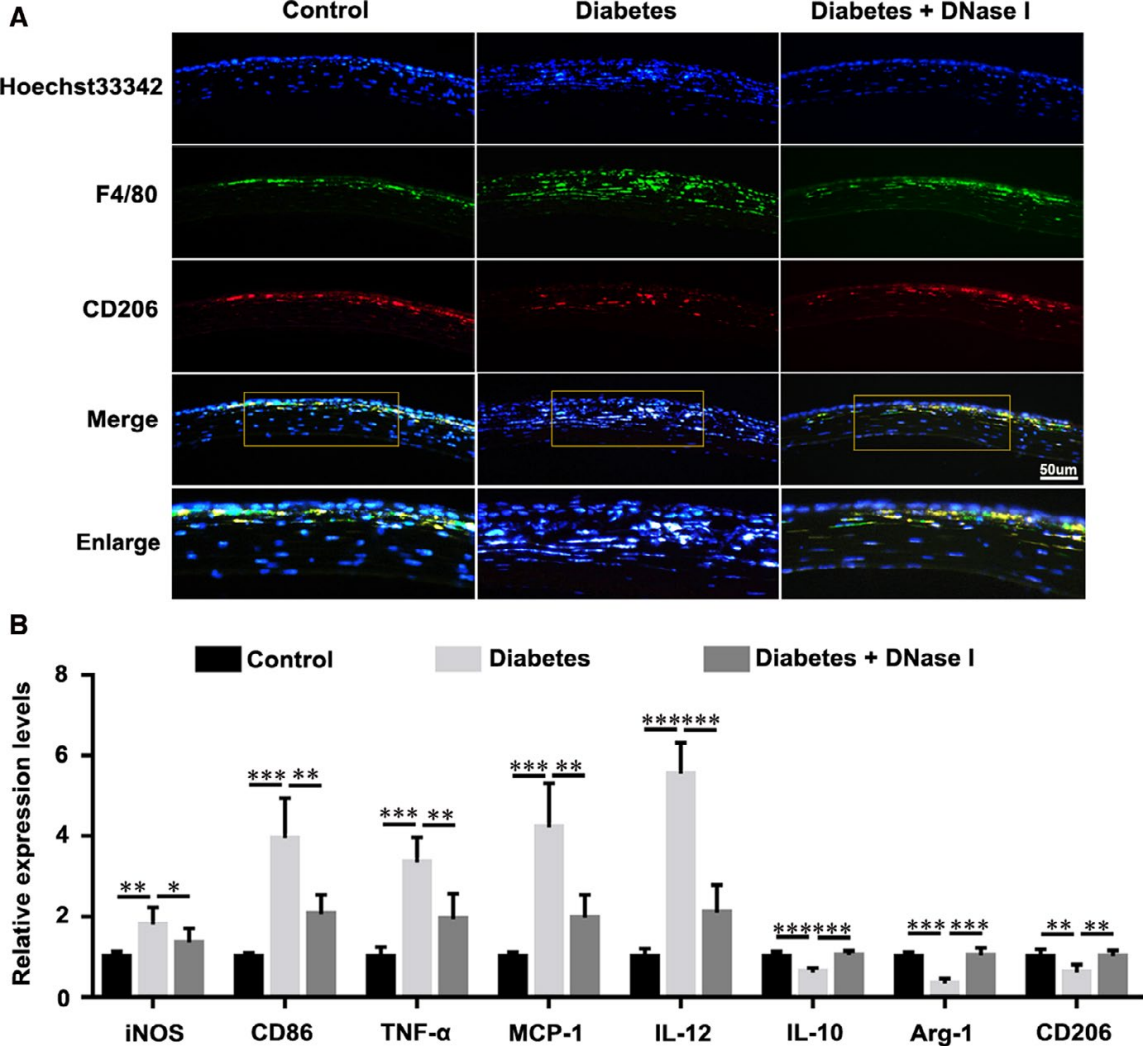
DNase I restored the resolution of corneal inflammation. A, Immunofluorescence staining was performed with the macrophage marker anti‐F4/80 (green fluorescence) and the M2 macrophage marker anti‐CD206 (red fluorescence) 48 h after removal of the corneal epithelium. B, mRNA expression levels of iNOS, CD86, TNF‐α, MCP‐1, IL‐12, IL‐10, arginase‐1 and CD206 (48 h after epithelial scrape) were analyzed by RT‐qPCR from the control and diabetic mouse corneas (n = 6). Data were given as the mean ± SD; *P < .05, **P < .01, ***P < .001

As a characteristic of inflammation resolution, the transition impairment of M1 to M2 macrophage phenotype was involved in the impaired wound healing in diabetic condition.^44^, ^45^, ^46^ Immunofluorescence staining results showed the enhanced staining of anti‐F4/80 (a macrophage marker) and anti‐CD206 (a M2 macrophage marker) in diabetic cornea, whereas topical application of DNase I remarkably reduced the infiltration of M1 macrophages and increased the proportion of M2 macrophages in diabetic cornea at 48 hours after injury (Figure 3A). In addition, qPCR results indicated the increased mRNA expressions of iNOS, CD86, TNF‐α, MCP‐1 and IL‐12 (which are specific for M1 subtype macrophages) and decreased mRNA expressions of IL‐10, arginase‐1 and CD206 (which are specific for M2 subtype macrophages) in diabetic cornea, whereas DNase I administration significantly rescued those expression alterations to a certain extent (Figure 3B).

**Figure 4:**
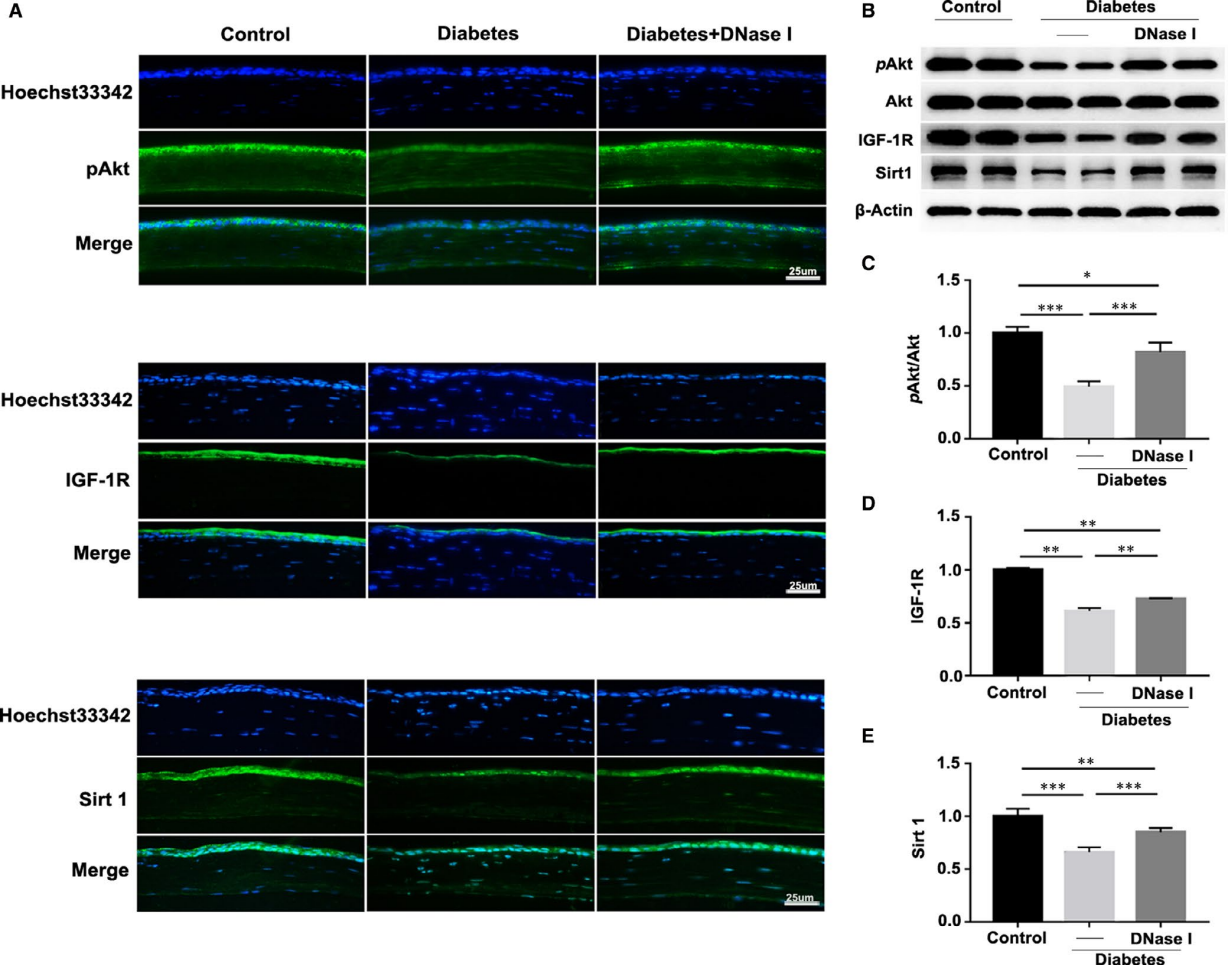
DNase I reactivated epithelial regeneration‐related signaling pathways. A, Immunofluorescence staining and (B) Western blot were used to examine the activation levels of epithelial regeneration‐related signaling pathways, including pAkt, IGF‐1R and Sirt1, in the regenerated corneal epithelium 48 h after injury. C‐E, The histogram showed the quantified results of the Western blot (n = 6). Data were given as the mean ± SD; *P < .05, **P < .01, ***P < .001

Previous studies have revealed the function of phosphorylated Akt (*p*Akt), IGF‐1R and Sirt1 in the promotion of corneal epithelial regeneration in diabetic mice.^9^, ^32^ Thereby, to explore the therapeutic mechanism of DNase I in corneal epithelial wound healing in diabetic mice, immunofluorescence staining and Western blot methods were used to examine the expression levels of *p*Akt, IGF‐1R and Sirt1 at 48 hours after epithelial removal. Representative images showed that topical application of DNase I significantly reversed the decreased staining of *p*Akt, IGF‐1R and Sirt1 in diabetic corneal epithelium (Figure 4A), which was further confirmed by the Western blot results showing that DNase I treatment efficiently rescued the expression levels of *p*Akt, IGF‐1R and Sirt1 in diabetic corneal epithelium, albeit not reaching the same expression levels in the age‐matched healthy control (Figure 4B, C; n = 6).

**Figure 5:**
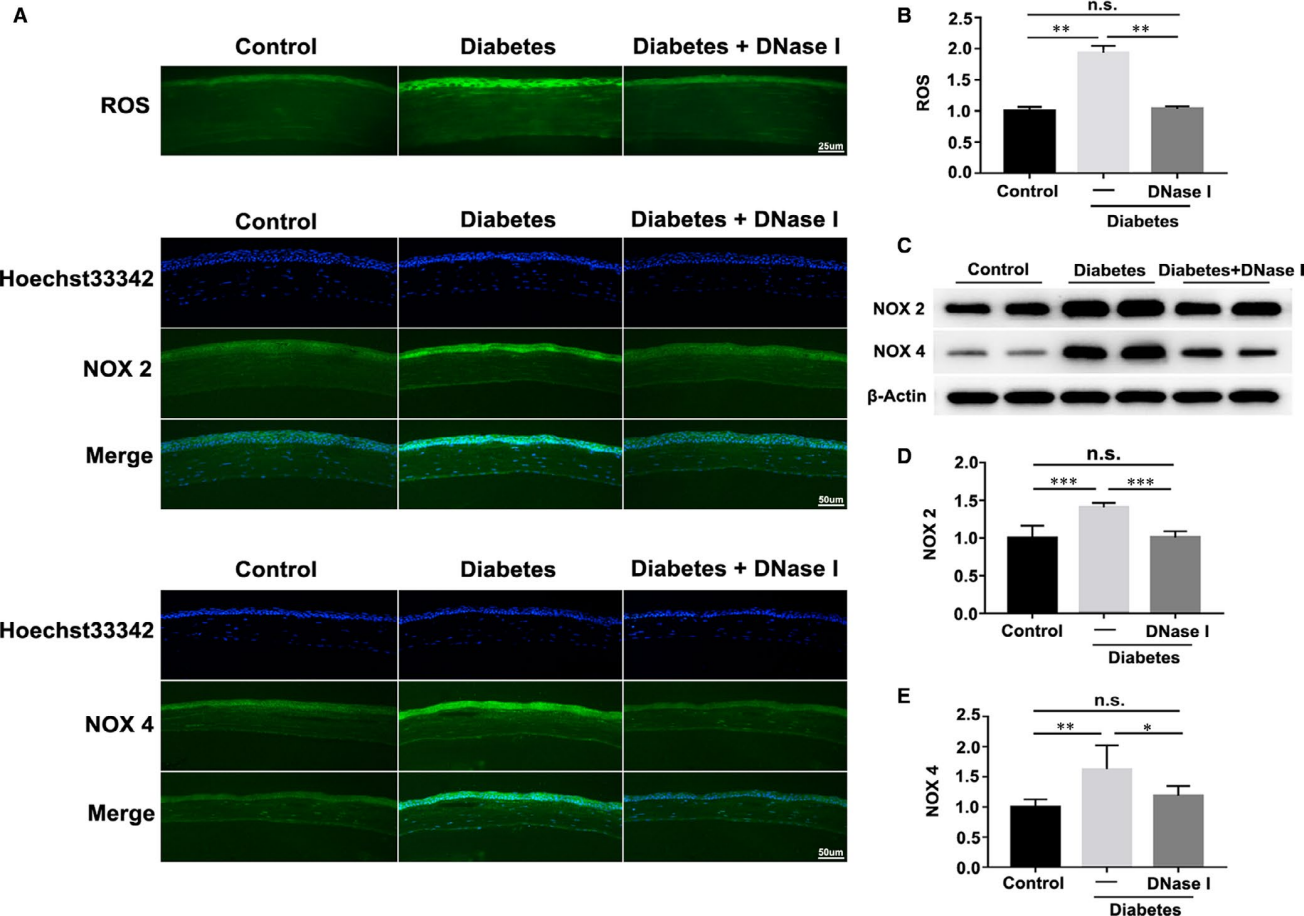
DNase I inhibited the increased ROS accumulation and NADPH oxidase 2/4 expression. A, Generation of reactive oxygen species (ROS) and expression of NADPH oxidase 2/4 were examined with immunofluorescence staining in healthy, diabetic and 5‐day DNase I‐treated diabetic corneal epithelia. B, Quantification of fluorescence intensity of ROS by ImageJ software (n = 3). C‐E, Corneas harvested from healthy and diabetic (with or without 5‐day DNase I treatment) mice were evaluated with Western blot to examine the protein levels of NADPH oxidase 2/4, and the quantified data of the Western blot results were shown (D, E; n = 6). Data were given as the mean ± SD; *P < .05, **P < .01, ***P < .001, n.s, not significant

Immunofluorescence staining results showed that increased ROS accumulation and NADPH oxidase 2/4 (NOX 2/4) staining occurred in diabetic corneal epithelium, whereas 5 days’ DNase I treatment significantly alleviated the accumulation of ROS (Figure 5A,B; n = 3) and the overexpressions of NOX 2/4 in diabetic corneal epithelium (Figure 5A). Consistently, results of Western blot showed that 5 days’ DNase I administration efficiently inhibited the overexpressions of NOX 2/4 in diabetic corneal epithelium, which almost fully recovered to the levels as in healthy control (Figure 5C,D; n = 6).

**Figure 6:**
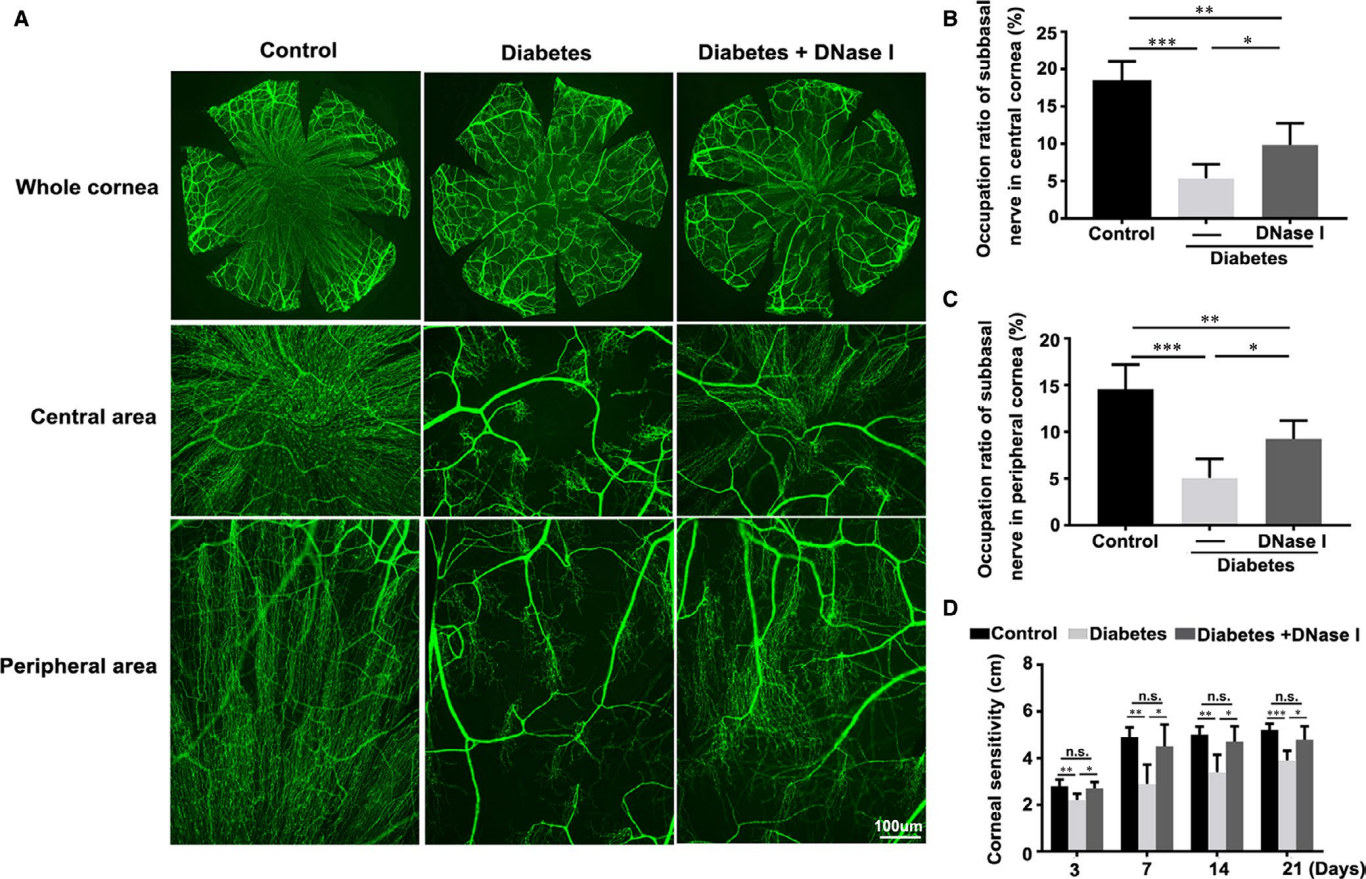
Effects of DNase I on the regeneration of diabetic corneal nerves and the restoration of mechanical sensation. A‐C, Twenty‐one days after injury, the renewing corneas were harvested and the regenerated corneal nerve fibres were examined with corneal whole‐mount staining, and the immunofluorescence intensity of central (B) and peripheral (C) nerve fibres at 21 d after injury was calculated with ImageJ software (n = 5). D, A Cochet‐Bonnet esthesiometer was used to test the mechanical sensitivity of the cornea in healthy, diabetic and DNase I‐treated diabetic mice at 3, 7, 14 and 21 d after the corneal epithelial removal (n = 5). Data were given as the mean ± SD; *P < .05, **P < .01, ***P < .001, n.s, not significant

To investigate the function of DNase I on the regeneration of injured cornea nerves in diabetic mice, corneal nerve morphology was measured after 21 days’ treatment of DNase I in mice with epithelial injury, and corneal mechanical sensation was examined at 3, 7, 14 and 21 days after DNase I treatment. Whole‐mount corneal nerve staining and analysis results showed that 21 days’ treatment with DNase I significantly improved the regeneration of diabetic corneal nerves (Figure 6A,B; n = 5), both in the central and peripheral areas of diabetic corneas (Figure 6B). Corneal mechanical sensitivity had significant improvement after 3 days’ DNase I treatment in diabetic mice with epithelial removal, and there was no statistic differences between DNase I‐treated diabetic mice and healthy controls at every test time‐point (Figure 6C; n = 5).

## 4 DISCUSSION

In this study, we found the eDNA‐containing NETs were formed during corneal epithelial wound healing both in normal and diabetic mice. Elimination of eDNA by DNase I promoted the regeneration of corneal epithelium in diabetic mice, proving that eDNA was a negative factor in the process of corneal epithelium regeneration in diabetic mice. DNase I and PAD4 inhibitors are two major methods for the intervention of NETs formation, but which one is better is still unknown. Although PAD4 inhibitors promoted corneal epithelial regeneration in diabetic mice at 48 hours, they had no therapeutic effect at 24 hours. DNase I had an earlier onset of action than PAD4 inhibitors, which not only reduced the chance of infection after diabetic corneal injury, but also showed that eDNA was a more important factor affecting the healing of diabetic corneal injury than NETs formation. Therefore, we chosed DNase I for further detailed research.

Results of this study showed significant improvements of corneal epithelial wound healing and corneal mechanical sensation restoration by DNase I administration in type 1 diabetic mice. Consistent with previous studies, the therapeutic mechanism of DNase I on ocular surface regeneration was related to the improvements of inflammation resolution, alleviation of ROS accumulation and activation of regeneration dependent signalling pathways. Detailedly, topical application of DNase I reactivated the regeneration‐related signalling pathways, including *p*Akt, IGF‐1R and Sirt1, and alleviated ROS accumulation and NOX 2/4 overexpression in diabetic mouse corneas. Furthermore, DNase I administration efficiently improved corneal nerve regeneration and sensation restoration in diabetic mice.

Oxidative stress is usually triggered by an imbalance between the formation of ROS and the antioxidant defence.^33^ With hyperglycaemia as a major aetiological factor, the occurrence of diabetes has a high association with increased oxidative stress.^34^ Previous studies have confirmed the effects of ROS accumulation in delaying wound healing of corneal epithelium,^8^ and attenuation of ROS accumulation serves as an important therapeutic target for the treatment of delayed corneal regeneration in diabetic condition.^27^, ^35^ The formation of NETs depends on the ROS produced by NADPH oxidase, which means that oxidative stress is the key to neutrophil NETosis.^36^, ^37^, ^38^ This present study consistently showed that DNase I application significantly alleviated the generation of ROS during diabetic corneal epithelial repair by suppressing the expression of NADPH oxidase. This was consistent with the results of Munafo DB's in vitro studies.^39^

Inflammation is a necessary self‐protective mechanism for organisms against the infection during unwarranted tissue damage, whereas the timely resolution of excessive inflammatory response is also required to maintain a healthy tissue‐repair environment. A series of biological processes involve in the resolution of inflammation, which include but not limited to the suppression of neutrophil infiltration and the production of pro‐inflammatory factors, promotion of macrophage phagocytosis and clearance of phagocytes.^40^ Furthermore, the transition impairment of M1 to M2 macrophage phenotype has been considered as a negative factor for impaired wound healing in diabetic condition. In short, M2 macrophages facilitate wound repair.^41^, ^42^, ^43^ Coincidently, this study indicated that the infiltration of neutrophils and the expression of pro‐inflammatory factors were suppressed after the administration of DNase I during corneal wound healing in diabetic mice, suggesting an underlying mechanism of DNase I function on diabetic corneal wound healing through, at least partly, attenuating NETosis. Moreover, DNase I also promoted the transition of M1 to M2 macrophage phenotype, which improved the clearance of inflammatory products and thereby the healing rate of corneal wound in diabetic mice. Similarly, previous study had confirmed that DNase I promoted wound repair in diabetic rats by alleviating the activation of NLRP3 inflammasome,^44^ suggesting that DNase I can not only reduce the inflammatory response of neutrophils, but also that of macrophages.

Decreased corneal sensation and corneal nerve fibre density serve as the typical clinical characteristics of diabetic keratopathy.^45^, ^46^ Our and other previous studies have already manifested that reduced corneal innervation is an critical influencing factor for the impaired corneal wound healing in diabetic condition.^47^ This work showed that topical DNase I administration promoted the regeneration of corneal nerve fibres and improved corneal sensation in diabetic mouse. There are few studies involving the relationship between eDNA and neurological diseases, except that NET markers are found in the brain parenchyma of Alzheimer's disease patients.^48^ Therefore, our results suggest that DNase I administration is a potential therapy for the treatment of impaired corneal epithelial innervation, even diabetic neuropathy caused by hyperglycaemia.

In summary, the present study suggests that topical application of DNase I improves corneal epithelial wound healing, accompanied by the improvement of inflammatory resolution, corneal nerve fibre regeneration and corneal sensation restoration in diabetic mice. The multiple function of DNase I in corneal epithelial and nerve regeneration provides a potential novel solution for the treatment of keratopathy in diabetic patients.

## CONFLICT OF INTEREST

The authors confirm that there are no conflicts of interest.

## AUTHOR CONTRIBUTIONS

Q. Zhou and L. Xie conceptualized the study. J. Zhang performed data analysis and visualization, and wrote the manuscript. J. Zhang conducted the wet‐lab experiments. Q. Zhou and L. Xie revised the manuscript. Y. Dai, C. Wei and X. Zhao provided technical assistance. All authors read and approved the final manuscript.

## Data Availability

All the data and materials generated and/or analysed during the current study are available.
